# Gold Inlays

**Published:** 1905-05-15

**Authors:** E. H. Allen

**Affiliations:** Freeport, Ills.


### GOLD INLAYS.

BY E. .H. ALLEN, D.D.S., FREEPORT, ILLS.

In my limited experience, I find the gold inlay to be a very valuable way of preserving the tooth when the conditions indicate such a method. I may say that gold inlays are indicated in large cavities in the bicuspids and molars, provided it does not result in an undue display of gold. Teeth may be saved by a gold inlay which, if filled with gold foil or amalgam, would best be treated by crowning. This may also be said of the porcelain inlay. To make a gold inlay that will properly fit the cavity it is intended to fill requires no more skill and practice than a good gold filling requires, and no less. The cavity, however, for an inlay requires an entirely different preparation than one for the gold filling. As all of you doubtless know, the cavity must have no undercuts to retain the inlay. In other words, the cavity must not in any way prevent the ready removal of the matrix. I prefer pure gold, at least No. 36 Brown & Sharp's gauge for the matrix.

Let us now suppose that we have a cavity prepared in a molar or bicuspid, either mesial or distal. I cut a piece of pure gold plate, the thickness before stated, large enough to line the cavity with plenty of surface to extend beyond the margins in all directions. Put this piece in place over the cavity, then with a good sized piece of spunk or wet cotton (I prefer spunk), push the gold to the bottom of the cavity; hold the gold in place by pressing upon the spunk with a broad-faced instrument, then with a burnisher work the gold against the margins of the cavity, working out any wrinkles in the gold. You now have a matrix that in a rough way conforms to the shape of the cavity. Remove and anneal, return to the cavity, place the spunk in position, then with a piece of cotton tape to hold matrix against margins of cavity burnish matrix perfectly against them. The matrix may be removed and margins trimmed to the outline of cavity, which is now plainly shown on the matrix. The matrix is now returned and refitted to cavity. It is then filled with a drop of hard, sticky wax to facilitate removal, the wax rendering it less liable to injury in handling. Matrix is then invested and when hard the investment is placed in the gas flame and heated. Let the wax burn out—20 or 22-carat gold solder is now flowed into the cavity until the right quantity is obtained to make a proper contour of the inlay. A very ingenious yet simple way of securing adaptation of the matrix to the cavity, for the discovery of which credit is due Dr. Clinton B. Helm, of Rockford, is the packing of soft gold foil into the matrix. This foil can be left in and solder flowed over it. This secures a very close adaptation. Dr. Helm also secures outline for contour and occlusion in case of occluso-approximal cavities in this way. When the matrix is in position take a piece of gold plate the same as matrix is made of, and conform it to shape as if for a simple matrix to be used to aid in inserting a filling. The top is cut so as to conform to the occlusion of upper or lower teeth, as the case may be. This is tied with ligature passing around the tooth; then the soft gold before mentioned is packed in place until proper contour is secured. I do not fill this cavity entirely with the gold foil, but only enough to satisfy myself that I have proper adaptation of matrix and cavity; then I drop some melted, hard, sticky wax upon the matrix. When this is hard, the ligature is cut and the entire piece is readily removed from the cavity. If care has been observed in properly adapting the part used to secure contour to the gold used as matrix, it will be found that the two parts are in actual contact. This piece then is invested, wax burned out, and filled with 22-carat solder. The heat should be applied to the under side of the investment. Thus solder will be drawn down and the inlay become one solid piece. After this, the edges should be properly trimmed to the margin of the cavity, and the inlay polished.

Before cementing to place, I have roughened the surface of the inlay with a cross-cut fissure bur in order that the cement may take a firmer hold of the inlay.

Another way of using the inlay is for cases of extensive wear of the occlusal surfaces of the molars and bicuspids, when it seems desirable to lengthen the bite. I can best describe this method by telling you how I treated a case about two years ago. A gentleman, about 38 years of age, had teeth which were practically immune from caries, but the occlusion was such that the incisors, upper and lower, struck directly against each other. There had been considerable loss of tooth structure as a result. I concluded that if the bite were lengthened, the wear would cease as far as the incisors were concerned. I therefore cut down the occlusal surface of the right inferior second molar to insure a flat surface and also to enable me to get a cusp of gold plate that would lengthen the bite enough to relieve the incisors. I then burnished and fitted 24-carat gold plate, about 36 gauge, over the flat top of the molar, leaving the edges flush. Then I drilled holes in each corner of the crown, perhaps 1-12 to 1-16 inch deep, diameter large enough to take a 20-gauge iridio-platinum wire, square wire preferred; then placing the cap of gold back in position, I found the location of the holes with a burnisher, punched holes through the plate, and drove the posts through, which had been cut to the requisite length; then flowed sticky wax over the top and cooled; removed and invested in wet asbestos and soldered posts to place. Then selected proper die for swaging the cusps for the case; 22-carat gold was used and the cusps filled with 22-carat solder. Placed the cap with posts soldered in position back on the tooth; then tried the cusps; if too thick to give the desired length, it was readily adjusted with a file. When the proper adjustment was secured placed hot sticky wax on the under side of the cusp and when cool enough not to run, placed on the gold cap which was in position on the tooth and directed the patient to bite. This placed cusps and caps in the proper relation to each other; removed this from the tooth as one piece, built wax over the edges where the solder was to flow, and invested; then heated up and filled the space between cap and cusps with 22-carat solder, finished and cemented in place. Next I took the left inferior second molar and treated the same way, then the first molars and bicuspids, until I had lengthened the bite as much as desired An excellent occlusion was in this way secured. I think I never performed a service for any patient who was better pleased and had as much comfort from the operation. All of these cusps are in position just as they were when cemented in place.—*Digest.*

